# Chemotherapy-Induced Neutropenia in HIV Positive Patients with Lymphoma: Comparison of Pegfilgrastim with Daily Filgrastim Administration

**DOI:** 10.4084/MJHID.2012.062

**Published:** 2012-10-03

**Authors:** Luciana Teofili, Immacolata Izzi, Eugenia Rosa Nuzzolo, Giancarlo Scoppettuolo, Lorenza Torti, Marianna Rossi, Katleen de Gaetano Donati

**Affiliations:** 1 Department of Hematology, Catholic University of Rome, Italy; 2 Department of Infectious Diseases, Catholic University of Rome, Italy

## Abstract

We retrospectively compared the incidence of neutropenia in two groups of HIV patients with lymphoma, who underwent chemotherapy supported by once-per-cycle administration of pegfilgrastim or by daily subcutaneous injection of filgrastim, respectively. Our findings indicate that pegfilgrastim and filgastrim produce similar results in preventing both neutropenia and febrile neutropenia.

## Introduction

HIV infection leads to an increased risk of non-Hodgkin’s lymphoma (NHL) and, to a minor extent, of Hodgkin’s lymphoma (HL). The concomitant administration of highly active antiretroviral therapy (ART) to chemotherapy (CT) has greatly improved the outcome of these diseases.^1^ Furthermore, the management of CT induced neutropenia with recombinant human granulocyte-colony stimulating factors (G-CSFs, i.e. filgrastim and lenograstim) rendered feasible intensive CT regimens, including dose-dense CT or high dose CT, also in HIV positive patients.^2^,^3^ A pegylated form of filgrastim is today available: actually, the addition of the polyethylene glycol molecule increases the serum half-life of filgrastim, resulting in its prolonged activity.^3^ Therefore, pegfilgrastim shows the advantage of a single injection per CT course as compared to repeated daily administration of filgrastim or lenograstim. The safety and efficacy of primary prophylaxis with all currently available G-CSFs in preventing febrile neutropenia (FN) and in supporting dose-dense therapy has been demonstrated in various malignancies and it is strongly recommended.^4^ However, a recent meta-analysis demonstrated that all currently available G-CSFs significantly reduces the incidence of FN in comparison with patients not receiving growth factors, whereas pegfilgrastim in particular appeared the most effective one.^4^ If so, the superiority of pegfilgrastim should be even more evident in the setting of AIDS associated tumors, since these patients have a particular high infectious risk. Nevertheless, to our knowledge, the efficacy of pegylated and non pegylated G-CSFs in HIV positive patients has never been investigated. In this retrospective study we compared the efficacy of pegfilgrastim and filgastrim in preventing neutropenia and fever in a series of HIV patients treated at our institution.

## Patients and Methods

The study population consisted of a series of 8 HIV positive patients with diagnosis of HL (n = 4) and NHL (n = 4), consecutively observed at the department of Infectious Diseases of the Catholic University of Rome between October 2005 and July 2006. These patients received CT regimens supported by once-per-cycle administration of pegfilgrastim. As a control group, 13 HIV positive patients (5 with HL and 8 with NHL) matched for age, diagnosis and CT, who had been previously followed at the same institution, were evaluated. All patients in the control group received CT supported by daily subcutaneous injection of filgrastim. Chemotherapy for HL consisted of ABVD^7^ in 3 patients with early stage HL, and of standard dose-BEACOPP^8^ or EBVP^7^ regimens in 6 patients with advanced stage-HL. Chemotherapy for NHL consisted of CHOP^7^ in 9 patients with diffuse large B-cell lymphoma and of Magrath protocol^9^ in 3 patients with Burkitt’s lymphoma. Considering that all patients were treated before 2006, none of them received Rituximab in association with CT. Both pegfilgrastim and filgrastim were administered 1 day after the completion of CT. In total, 23 courses of CT were evaluated in the pegfilgrastim group and 75 courses in the filgrastim group. The efficacy of pegfilgrastim and of filgrastim was assessed by evaluating the incidence of severe neutropenia (defined as neutrophil count less than 0.5 × 10^9^/L), the number of FN episodes and the number of positive blood cultures occurred among all recorded CT courses. Statistical comparison of continuous variables was performed by the Mann-Whitney U-test. Comparison of categorical variables was performed by chi-square statistic, using the Fisher’s exact test. P values <0.05 were considered statistically significant.

## Results

Clinical and laboratory findings of patients are shown in Table 1. No differences were found between pegfilgrastim or filgastrim groups of patients for sex and age distribution, stage of disease, Type of CT, and hematological parameters (Table 1). Moreover, the number of CD4+ cell count at diagnosis (evaluated as continuous variable) and the percentage of patients showing CD4+ cell count less than 200/μL were similar in both groups (Table 1). A similar proportion of patients in pegfilgrastim and filgrastim groups received ART in combination with CT (Table 1). Table 2 shows the efficacy end points observed in pegfilgrastim- as compared to filgrastim-supported CT courses. Severe neutropenia was observed in 8 out of the 23 CT courses analyzed in the pegfilgrastim group, and in 32 out of the 75 CT courses analyzed in the filgrastim group (p=0.63, Table 2). The median duration of neutropenia was 5 days in both groups (p=0.80, Table 2). FN was recorded in 5 cases in pegfilgrastim group and in 27 cases in filgrastim group (p=0.14, Table 2). Accordingly, no differences were found between pegfilgrastim and filgrastim-supported-CT in the incidence of positive blood cultures (p=1.00, Table 2).

## Discussion

To compare the efficay of pegylated and non-pegylated G-CSFs in the setting of HIV associated malignancies is an interesting issue to address. According to evidence-based clinical practice, primary prophylaxis with G-CSFs is recommended for CT regimens exceeding the 20% threshold of FN risk^4^,^10^ and in dose-dense CT schedules.^11^,^12^ The existence of concomitant conditions predisposing to prolonged neutropenia, such as other myelotoxic therapies or bone marrow involvement by lymphoma cells, may help to identify which patients are most likely to benefit from prophylactic G-CSFs.^4^,^10^ In this respect, HIV positive persons undergoing CT for lymphoma represent a patients population at high risk for infection and FN. Actually, the diagnosis of lymphoma and HIV infection frequently occurs simultaneously^1^ and lymphoid tumors often arise in most immunocompromised HIV patients.^13^ In addition, the majority of HIV infected patients receives antiretroviral drugs and CT in combination: importantly, not only ART can have a direct hematologic toxicity^14^ but, in some instances, it can affect the metabolism of antiproliferative drugs so enhancing their bone marrow toxicity.^15^ Finally, lymphomas occurring in HIV positive patients, involve bone marrow more frequently than in general people.^16^ Our study shows that pegfilgrastim and filgrastim exhibit similar efficacy in controlling both CT-induced neutropenia and FN episodes in HIV positive patients with HL and NHL. Notwithstanding the worth of our findings is limited by the retrospective design of the study, they can suggest some considerations. While filgrastim and lenogratim are administered by a course of daily injections, pegfilgrastim posses the important advantage of once-per-cycle administration. This is an important aspect considering that several data from low level studies derived from audit data and clinical practice suggests that some patients receive suboptimal daily G-CSFs^4^ and the use of pegfilgrastim prevents this problem. Likewise, however, it is important to consider the cost of care is rising progressively, and that it is partly due to unnecessary use of health care resources. To this purpose, the American Society of Clinical Oncology has recently stated, among other opportunities to improve care and reduce costs, that G-CSFs should be used only when the risk of FN, secondary to a recommended chemotherapy regimen is approximately 20% and equally effective treatment programs that do not require white cell stimulating factors are unavailable.^17^ In line with these recommendation, pegfilgrastim support should be mainly tailored to a cost-effective care provision.^11^,^12^ Nevertheless, in HIV positive patients, the simplified one-per cycle injection could result also in the reduced risk of infection by health care operators. This important topic should be considered an additional point to be clarified in studies performed on HIV infected patients.

## Figures and Tables

**Table 1:** Clinical and laboratory findings of HIV positive patients with Hodgkin lymphoma (HL) and non Hodgkin lymphoma (NHL), receiving pegfilgrastim or filgrastim, respectively.

	Pegfilgrastim	Filgrastim	
		
Patient n°	8	13	*p*
**NHL/HL**	4/4	8/5	*0.67*
**Males/Females**	7/1	8/5	*0.33*
**Age(years): Mean**	45,2	41,8	*0.66*
**Median (Range)**	45,5 (28–62)	44 (28–51)	
**WBC × 10^9^/L : Mean**	4.4	4.7	*0.84*
**Median (Range)**	4.5 (3.6–5.1)	4.3 (0.8–8.7)	
**Hb g/dl: Mean**	11	11,5	*0.68*
**Median (Range)**	11,3 (9–13,9)	11,1 (9,3–13,3)	
**PLT × 10^9^/L: Mean**	226	147	*0.48*
**Median (Range)**	257 (110–297)	107 (19–296)	
**Stage**			*0.11*
**I/II**	1	2	
**III/IV**	7	11	
**B symptoms (%)**	6 (75 )	10 (77)	*1*
**Chemotherapy**			*0.45*
**CHOP**	3	6	
**Magrath**	1	2	
**ABVD**	1	2	
**BEACOPP**	3	1	
**EBVP**	-	2	
**HAART (%)**	7 (87%)	12 (92 %)	*1*
**CD4+ cells < 200/**μ**L (%)**	4 (50)	8 (62)	*0.67*

**Table 2:** Incidence of severe neutropenia (neutrophils < 0,5 × 10^9^/L), febrile neutropenia and positive blood cultures in 23 chemotherapy courses supported by pegfilgrastim and in 75 chemotherapy courses supported by filgrastim.

	Pegfilgrastim		Filgrastim		
		
Total courses	23		75		*p*

	*n°*	*%*	*n°*	*%*	
**Grade IV neutropenia**					
**n. of episodes**	8	35	32	43	*0.63*
**days: median (range)**	5 (2–7)		5 (1–20)		*0.80*
**Febrile neutropenia**	5	22	27	36	*0.32*
**Positive blood cultures**	3	(13)	9	12	*1*
